# Regrowing axons with alternative splicing

**DOI:** 10.7554/eLife.18707

**Published:** 2016-07-15

**Authors:** Nicholas J Kramer, Aaron D Gitler

**Affiliations:** 1 Department of Genetics and the Neurosciences Graduate Program, Stanford University School of Medicine, Stanford, United Statesnjkramer@stanford.edu; 2 Department of Genetics, Stanford University School of Medicine, Stanford, United Statesagitler@stanford.edu

**Keywords:** post-transcriptional regulation, UNC-75, PNS regeneration, DRG, CUGBP, neurite outgrowth, *C. elegans*, Mouse

## Abstract

The regeneration of axons relies on a previously unknown mechanism that involves the regulation of alternative splicing by CELF proteins.

**Related research article** Chen L, Liu Z, Zhou B, Wei C, Zhou Y, Rosenfeld MG, Fu X-D, Chisholm AD, Jin Y. 2016. CELF RNA binding proteins promote axon regeneration in *C. elegans* and mammals through alternative splicing of Syntaxins. *eLife* **5**:e16072. doi: 10.7554/eLife.16072

**Image** Some mutant worms have problems regenerating axons

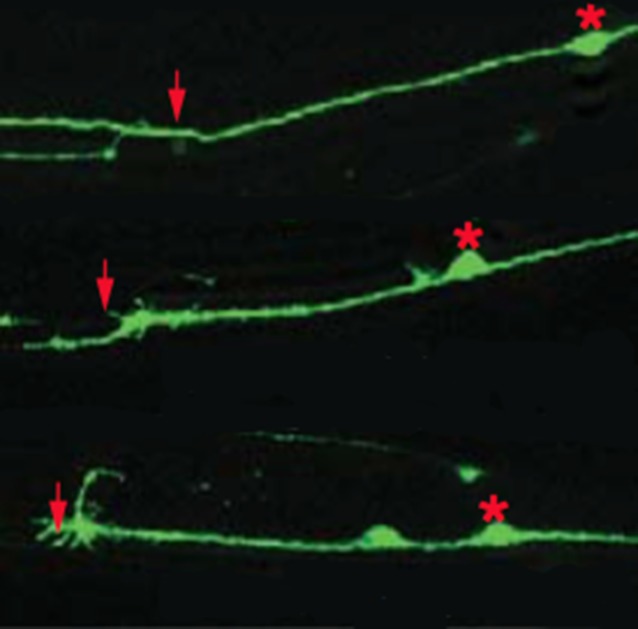

Injuries to neural circuits can mean that nerve fibers called axons need to be regrown. However, some nerve cells are better at regenerating than others, and understanding why this is so may open the door to new treatments for traumatic nerve injuries (He and Jin, 2016).

Researchers have studied the suites of genes that are activated in injured nerve cells for decades (Benowitz et al., 1981; Gervasi et al., 2003). However, relatively little is known about the post-transcriptional aspects of the regeneration process (that is, about what happens after the genes’ DNA has been transcribed to make molecules of messenger RNA (mRNA)). RNA binding proteins, for example, can influence almost every step of the life cycle of an mRNA (Vuong et al., 2016). This includes a process that many mRNAs undergo called alternative splicing, which allows several different proteins to be produced from the instructions encoded in a single gene. Furthermore, disrupted RNA metabolism and changes to alternative splicing underlie a wide spectrum of diseases in humans, including amyotrophic lateral sclerosis and other neurological disorders (Scotti and Swanson, 2015).

One family of RNA binding proteins called CELF proteins regulates alternative splicing in the brain and affects both the development and activity of neurons (Ladd, 2013). Now, in eLife, Yishi Jin and colleagues at the University of California, San Diego – including Lizhen Chen as first author – report how one of these proteins encourages axons to regrow in both worms and mice (Chen et al., 2016). To begin, Chen et al. saw that, unlike wild-type worms, worms lacking a CELF protein called UNC-75 had problems regenerating axons that had been cut with a laser. They then showed that UNC-75 acts within individual neurons to promote the regeneration of the axon belonging to that neuron (i.e. it acts cell autonomously).

Chen et al. reasoned that if, they could identify which RNAs binds to, it might give them some clues as to this protein’s function. They turned to a high-throughput genomics approach called CLIP-seq and generated a list of target RNAs from worm neurons (Licatalosi et al., 2008; Zisoulis et al., 2010). The majority of these UNC-75 binding sites were found within the introns of genes, which suggested that alternative splicing plays a major role in axon regeneration. Chen et al. then concentrated on a subset of the target RNAs and eventually focused on one that encodes a protein called UNC-64 or Syntaxin.

The mRNA for UNC-64/Syntaxin is spliced differently in neurons and non-neuronal tissues (Saifee et al., 1998), and Chen et al. suggest that UNC-75 promotes the expression of the isoform of Syntaxin that is found predominantly in neurons (called UNC-64A). Further studies with mutant worms led them to conclude that *unc-75* mutants have problems regenerating their axons largely because they produce less UNC-64A. This result is quite remarkable. Of the hundreds of RNAs identified as targets of UNC-75, it appears that one – that is to say, UNC-64A – is the key to promoting nerve regrowth following an injury.

Chen et al. extended their analyses from worms to mammals, and found that two CELF proteins from mice, including CELF2, could partially complement the function of UNC-75 during axonal regeneration in worms. CELF2 is the mammalian version of UNC-75, and mice that did not produce CELF2 in their nervous system had severe defects in axon regeneration. Finally, another round of CLIP-seq in mouse cells revealed that, similar to UNC-75 in worms, CELF2 binds to many Syntaxin mRNAs and can regulate the alternative splicing of two mammalian Syntaxin genes.

These experiments with worms and mice suggest that the CELF proteins are important players in axon regeneration, possibly via the post-transcriptional regulation of isoforms of Syntaxin. These new data pose a further question: do these Sytnaxin proteins directly promote axon regeneration, and what are the roles for the distinct isoforms during this process? Chen et al. present data demonstrating that this newly discovered CELF/Syntaxin pathway might control the ability of an axon to extend (Figure 1). Notably, Syntaxin proteins have been implicated in expanding the plasma membrane before (Bloom and Morgan, 2011; Darios and Davletov, 2006). However, if these mechanisms rely on tipping the balance between the alternatively spliced isoforms of Syntaxins remains to be explored.

**Figure 1.**
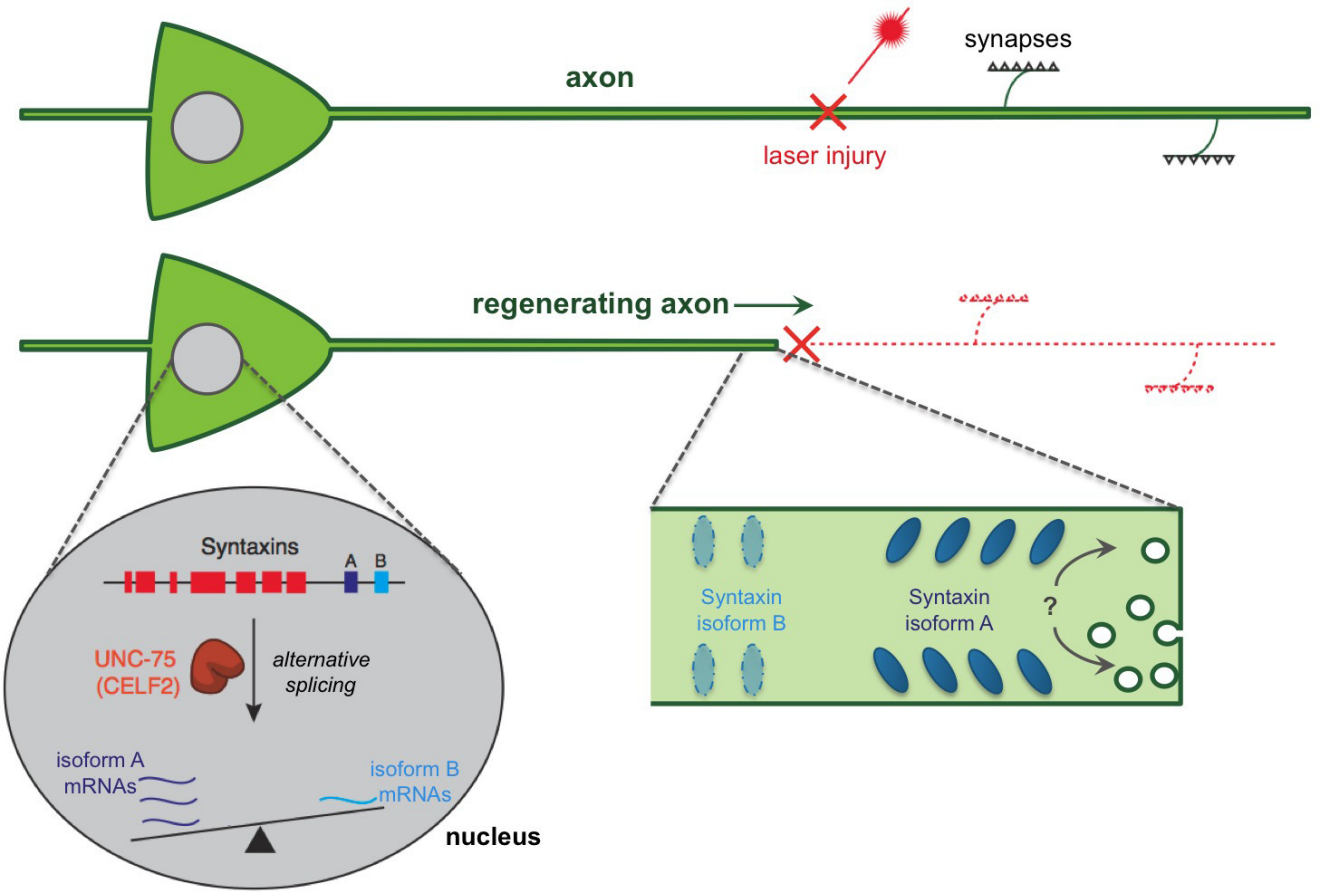
A regulatory pathway governing axon extension during neural regeneration. Cutting axons with a laser allows the regeneration process to be studied. Chen et al. report that an RNA binding protein (called UNC-75 in worms and CELF2 in mice) promotes axon regeneration by controlling the alternative splicing of an mRNA that encodes a protein called Syntaxin. This process takes place in the nucleus of the nerve cell and essentially ‘tips the balance’ of alternative splicing towards an isoform of Syntaxin that is found predominantly in neurons (shown in dark blue and labeled “A”), and away from an isoform that is usually found in non-neuronal tissues (light blue; “B”). In fact, UNC-75 tips the balance so strongly that the Syntaxin isoform B was not found in neuronal tissues unless the gene for UNC-75 was mutated. It is not clear how the Syntaxin isoforms contribute to regeneration. It has been proposed that the Syntaxin proteins promote extra membrane to be added to the axon, possibly aiding in extending the axon after an injury (indicated by a “?”). It is also possible that different Syntaxin isoforms segregate into distinct regions of the axon membrane leading to the different functions of these isoforms.

Finally, the CELF2 protein is not new to the spotlight. In fact, CELF2 has already been investigated as a therapeutic target in a neuromuscular disease called spinal and bulbar muscular atrophy, and lowering CELF2 levels can improve symptoms in a mouse model of this disorder (Miyazaki et al., 2012). What is new is that Chen et al. have provided a great resource to better understand which genes are targeted by CELF2. This new resource will also allow other researchers in the field to explore how regulating alternative splicing might factor into complex neurological diseases and neural repair strategies.
